# Marine heatwaves exacerbate climate change impacts for fisheries in the northeast Pacific

**DOI:** 10.1038/s41598-020-63650-z

**Published:** 2020-04-21

**Authors:** William W. L. Cheung, Thomas L. Frölicher

**Affiliations:** 1 Changing Ocean Research Unit, Institute for the Oceans and Fisheries, The University of British Columbia, Vancouver, B.C. V6T 1Z4 Canada; 2 Climate and Environmental Physics, Physics Institute, University of Bern, Bern, Switzerland; 3 Oeschger Centre for Climate Change Research, University of Bern, Bern, Switzerland

**Keywords:** Ecology, Ecology, Environmental sciences, Ocean sciences

## Abstract

Marine heatwaves (MHWs) have occurred in all ocean basins with severe negative impacts on coastal and ocean ecosystems. The northeast Pacific 2013–2015 MHW in particular received major societal concerns. Yet, our knowledge about how MHWs impact fish stocks is limited. Here, we combine outputs from a large ensemble simulation of an Earth system model with a fish impact model to simulate responses of major northeast Pacific fish stocks to MHWs. We show that MHWs cause biomass decrease and shifts in biogeography of fish stocks that are at least four times faster and bigger in magnitude than the effects of decadal-scale mean changes throughout the 21st century. With MHWs, we project a doubling of impact levels by 2050 amongst the most important fisheries species over previous assessments that focus only on long-term climate change. Our results underscore the additional challenges from MHWs for fisheries and their management under climate change.

## Introduction

Marine heatwaves (MHWs) - persistent extremely warm ocean temperatures - are already impacting ecosystems worldwide^1–5^. Impacts from MHWs include range shifts of marine fishes and invertebrates^6–9^, bleaching of coral reefs^2^, mass mortality of kelp forest^4,10^ and other coastal vegetation^11^ and reduction in reproductive success and survivorship of marine animals^12^. Long-term ocean warming since the early 20th century due to human-induced increase in greenhouse emissions has led to widespread increases in MHW frequency, intensity and duration^13^. Globally, the frequency of MHWs has been doubled since 1982^14^, and is projected to increase further under continued global warming^5,14,15^.

In 2013, a large MHW in the northeast Pacific appeared off the coast of Alaska and subsequently expanded south to Baja California. This specific MHW, commonly known as the “Blob”^16^, persisted through to the end of 2015 and was the largest MHW globally since 1982^17^ with sea surface temperature (SST) anomalies of over 6 °C. This warm Blob affected ecosystems from the California Current in the South to the Gulf of Alaska and the Bering Sea in the North^9,18–20^. The anomalously high temperature enhanced the stratification of the upper ocean, leading to a decrease in nutrient supply to the surface ocean and causing a decrease in net primary production and community production^21,22^. Observational studies have reported ecological changes in the Northeast Pacific region, such as shifts in the horizontal and vertical distributions of marine species^8,19^, as well as changes in pelagic micronekton and macrozooplankton communities and their species richness^18,23^. Such changes impacted also human activities such as fisheries^19^. Towards the end of 2019, a new MHW has emerged in the North Pacific^24^, raising concerns that a similar MHW as the Blob in 2013–2015 may reappear in the near future. Due to the already low numbers of Pacific cod (*Gadus macrocephalus*) and the potential reappearance of the Blob, the United States’ federal cod fishery in the Gulf of Alaska closed for the 2020 season as a precautionary measure^25^. The fisheries closure underscores the potential high impacts of such MHWs not only on marine ecosystems, but also on social-economic systems such as fisheries.

Simulating ecological changes of fish stocks and fisheries using modelling approaches can help elucidate and attribute the relative contribution of MHWs to observed changes in ecosystems^26^ and assess future ecological risks under alternative scenarios of climate change^27^. However, projections of ecological impacts of MHWs have focused mainly on sensitive biogenic habitats such as coral reefs and intertidal systems^28,29^. In contrast, previous impact assessments on fish stocks and fisheries focused mainly on decadal-scale changes in mean conditions under climate change while the additional impacts of MHWs are more uncertain. Improved understanding of the futures of living marine resources will help inform dependent human communities, sectors and governance institutions to develop more effective climate-adaptation and risk-reduction measures.

**Figure 1:**
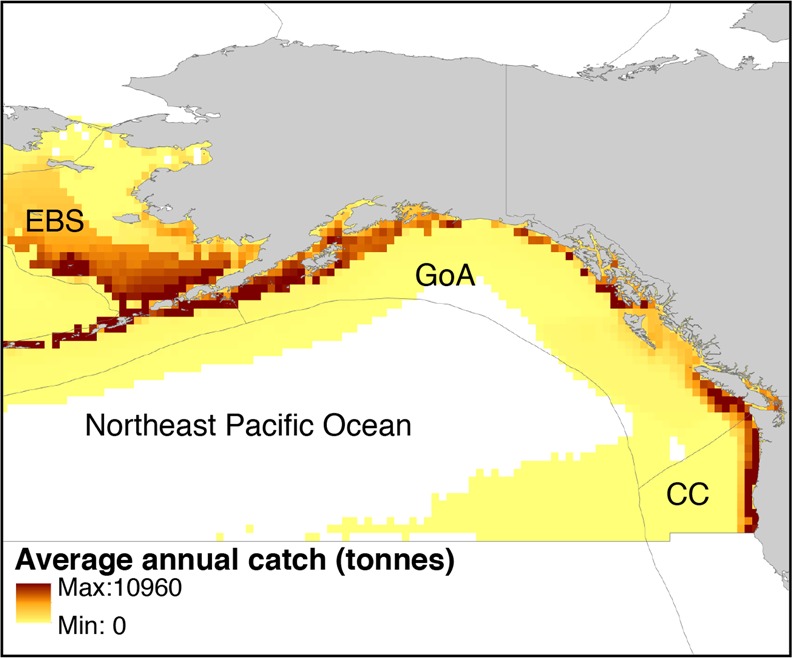
Average annual fisheries catches of the 22 studied fish stocks from 1981 to 2015 in the northeast Pacific Ocean and the three large marine ecosystems therein. Catches data were obtained from the Sea Around Us fisheries database (www.seaaroundus.org)^55^. The boundary of the northeast Pacific Ocean is based on the United Nations’ Food and Agriculture Organization Statistical Area 67. The large marine ecosystems include: EBS - Eastern Bering Sea, GoA - Gulf of Alaska, and CC - California Current.

Here, we aim to test the hypotheses that MHWs will add to the impacts of changes in mean ocean conditions under climate change, leading to additional anomalous shifts in biomass, distribution and potential catches of fish stocks in the northeast Pacific regions. Previous studies have applied species distribution models to project the effects of changing long-term mean ocean conditions on spatial distribution, abundance, community structure and the potential biomass production of fishes and invertebrates in this region^27,30,31^. In this study, we extend such modelling approaches to examine the consequences of MHWs on fish stocks and fisheries. Our analysis focused on the northeast Pacific Ocean and the Large Marine Ecosystems (LMEs) therein where most fishing took place (Fig. 1). We used the United Nations’ Food and Agriculture Organization (FAO) Statistical Area (Area 67) to delineate the northeast Pacific region. This region includes three LMEs^32^: (a) Eastern Bering Sea, (b) Gulf of Alaska and (c) California Current. We examine the additional risk of MHWs on fish distribution[biomass] and and potential fisheries catches and explore whether and how the projected impacts of MHWs add to the decadal scale changes in mean ocean conditions. We also discuss the implications of the findings for ecosystem-based fisheries management.

**Figure 2:**
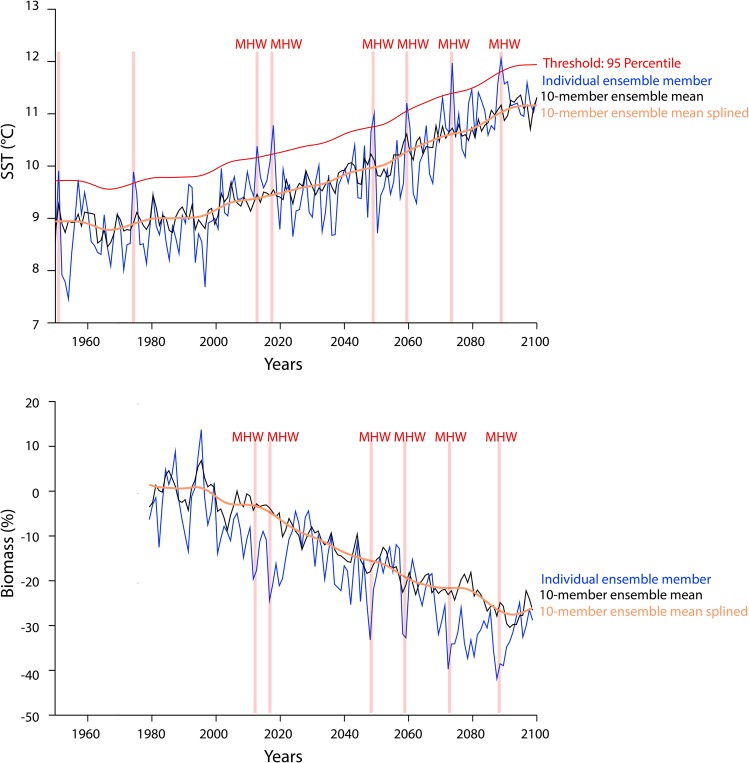
Schematic diagram explaining the characterization of Marine Heatwaves (MHWs) and their impacts on fish stocks. (**a**) The characterization of MHWs based on outputs from the 10 ensemble member projections of the GFDL ESM2M and (**b**) their impacts on biomass of the sockeye salmon (*Oncorhynchus nerka*) in the Gulf of Alaska large marine ecosystem. The red vertical bars in both panels indicate MHW events. Biomass changes are given as changes relative to 1986 to 2005.

We analyzed projected changes in annual mean Sea Surface Temperature (SST) in the northeast Pacific Ocean using a 10-member ensemble simulation of the Earth system model version 2 developed at the Geophysical Fluid Dynamics Laboratory (GFDL ESM2M^33–35^; see Materials and Methods). Each ensemble simulation was run over the 1950–2100 period under the same external forcing of historical changes before 2005 and Representative Concentration Pathway 8.5 (RCP8.5) thereafter. The RCP8.5 scenario represents a pathway of greenhouse gas concentrations for which radiative forcing reaches approximately 8.5 Wm^−2^ by 2100. We simulated changes in abundance and distributions of exploited fish stocks that are highly important to fisheries in the northeast Pacific region (Fig. 1) We included a total of 22 fish species that were reported in the fisheries statistics in the northeast Pacific region (www.seaaroundus.org). These species were important to fisheries in this region as they contributed up to 80% of the total observed catches from 2006 to 2015 (www.seaaroundus.org). We used the dynamic bioclimate envelope model (DBEM)^36,37^, which is a spatially-explicit species distribution-population dynamic model, to simulate dynamical changes in biomass, and potential fisheries catch for each species on a 0.5° latitude × 0.5° longitude grid of the world ocean (see Methods for details). To identify MHWs, we calculated anomalies between the annual mean SST simulated by each of the individual 10 ensemble members and the ensemble-averaged SST (Fig. 2). We calculated four impact indicators to examine the ecological responses of fish stocks and their implications for fisheries during a MHW. These indicators are: (1) total biomass, (2) latitudinal centroid (average of the coordinates of grid cell weighted by the species’ biomass), (3) depth centroid (average of bathymetry of grid cell weighted by the species’ biomass), and (4) maximum catch potential [catch at fish stock-specific fishing mortality rate (F) that achieves maximum sustainable yield (MSY) i.e., F = F_MSY_].

## Results

**Figure 3:**
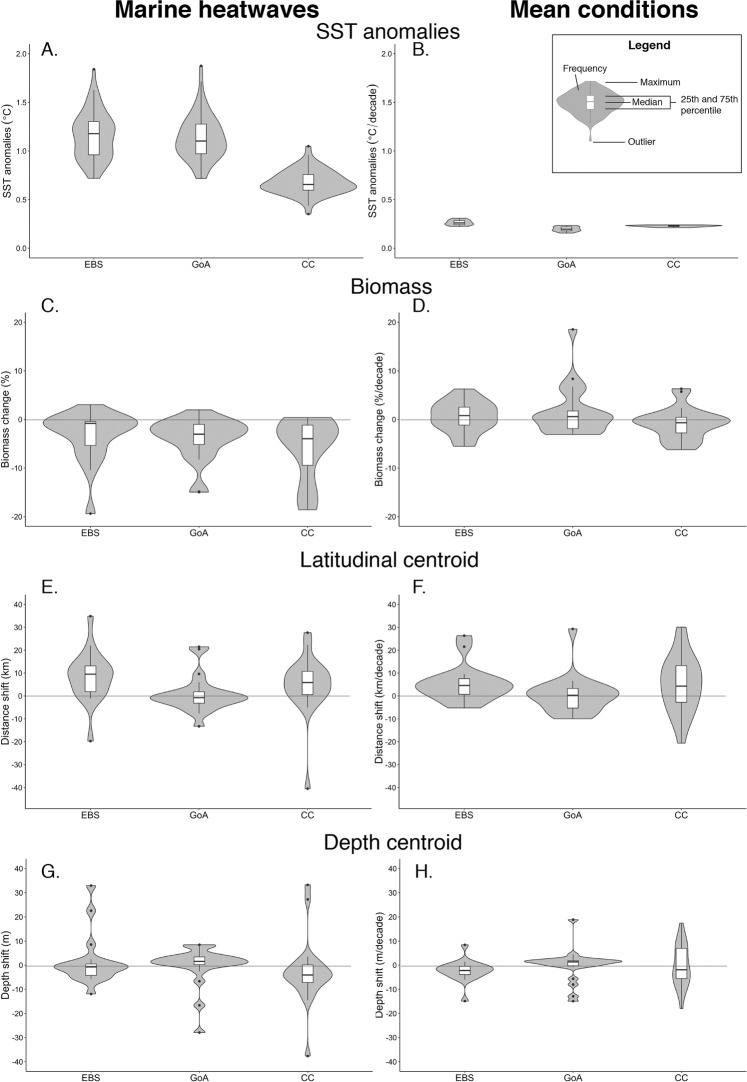
Projected changes in sea surface temperature (**A**,**B**) and the impact indicators (biomass, latitudinal centroid and depth centroid; **C**–**H**) of the 22 studied fish stocks in the three large marine ecosystems (Eastern Bering Sea - EBS, Gulf of Alaska - GoA, California Current - CC) of the northeast Pacific during MHWs (left panels) and due to long-term climate change from 1981 to 2100 (right panels). Changes in sea surface temperature and impact indicators during MHWs were expressed relative to the spline-smoothed ensemble-mean of the same time period. In contrast, long-term climate changes were calculated from linear regressions over the 1981–2100 period, with the rate of change (slope of the regression) expressed as change per decade. Negative depth centroid shifts in G-H indicate species’ average distribution that were projected to be deeper.

We identified amongst the ten ensemble member simulations in total 149 MHWs in any of the three LMEs from 1981 to 2100. During these MHWs, the SST anomalies (i.e. mean annual intensity) are on average 0.99 °C (5^th^ to 95^th^ percentile = 0.55–1.49 °C) higher than the ensemble-mean SST (Fig. 3A). In comparison, the simulated average rate of SST change across the LMEs is 0.23 ± 0.04 °C (standard deviation) per decade (Fig. 3B). Thus, the average MHW SST anomalies, which are estimated annually, are about four times the mean warming per decade in the northeast Pacific LMEs. The intensity of MHWs is higher in the high latitude LMEs, i.e., Eastern Bering Sea and Gulf of Alaska, relative to that in California Current (Fig. 3A), because the SST variability is larger in Eastern Bering Sea and Gulf of Alaska than in California Current. Since we focused on annual means in SST, the modeled SST anomalies in all three LMEs are in general smaller than the observed peak SST anomalies during the Blob.

The biomass of the 22 exploited fish stocks in the three northeast Pacific LMEs was projected to decrease by a median of −2.8% (5^th^ to 95^th^ percentile = −17.1 to 0.4%) during MHW events (Fig. 3C), with maximum decreases of up to 20%. Changes in maximum catch potential levels were almost the same as changes in biomass (Fig. S4–6). The biomass (and maximum catch potential levels; not shown) of almost all fishes in the California Current fish stocks were lower during MHWs for the majority of the fish stocks (median = -4.0%, 5th to 95th percentile from −18.3 to 0.0%). The direction of impacts of MHW on fish stock biomass in the Eastern Bering Sea (−0.8%, from −11.7 to 1.1%) and Gulf of Alaska (−3.0%, from −14.5 to 0.2%) were slightly more variable. These general decreases in biomass during MHWs added to the long-term climate change-induced changes in biomass. The decadal mean changes in biomass were projected to be more variable in the direction of changes than changes during the MHWs years across the LMEs (Fig. 3D). For the species with negative responses during MHWs, the decreases in biomass during MHWs were several factors higher than the rate of biomass decrease per decade from 1981 to 2100 under RCP8.5.

We also projected signature of MHWs on the biogeography of exploited fish stocks in the northeast Pacific (Figs. 3E,G). 70% of the fish stocks showed a poleward shift in the latitudinal distribution centroids during MHWs (Fig. 3E). The direction of shifts in the Gulf of Alaska were projected to be more variable across species (median = −0.76 km, 5th to 95th percentile from −7.6 to 19.9 km) than those in Eastern Bering Sea and California Current, with around half of the species shifting southward. On average, fish assemblages in Eastern Bering Sea and California Current were projected to shift poleward at a rate of 9.5 km (−4.0 to 23.9 km) and 5.8 km (−5.1 to 22.0 km) per year, respectively, for each MHW event, with maximum shifts of over 30 km relative to the mean distribution. The pattern and magnitude of the latitudinal shifts of the fish assemblages were similar to the average decadal-scale shifts under climate change (Fig. 3F). Bathymetric shifts with MHWs were projected to vary more substantially across the stock-ensemble members, particularly in Eastern Bering Sea (−0.8, −6.7 to 24.1 m) and California Current (−4.1, −14.4 to 26.1 m) compared to Gulf of Alaska (1.6, −16.1 to 8.1 m); note negative values indicate shift to deeper waters). The pattern of shifts in depth centroids of fish assemblages were generally consistent between MHWs and long-term decadal-scale mean changes (Fig. 3G,H).

**Figure 4:**
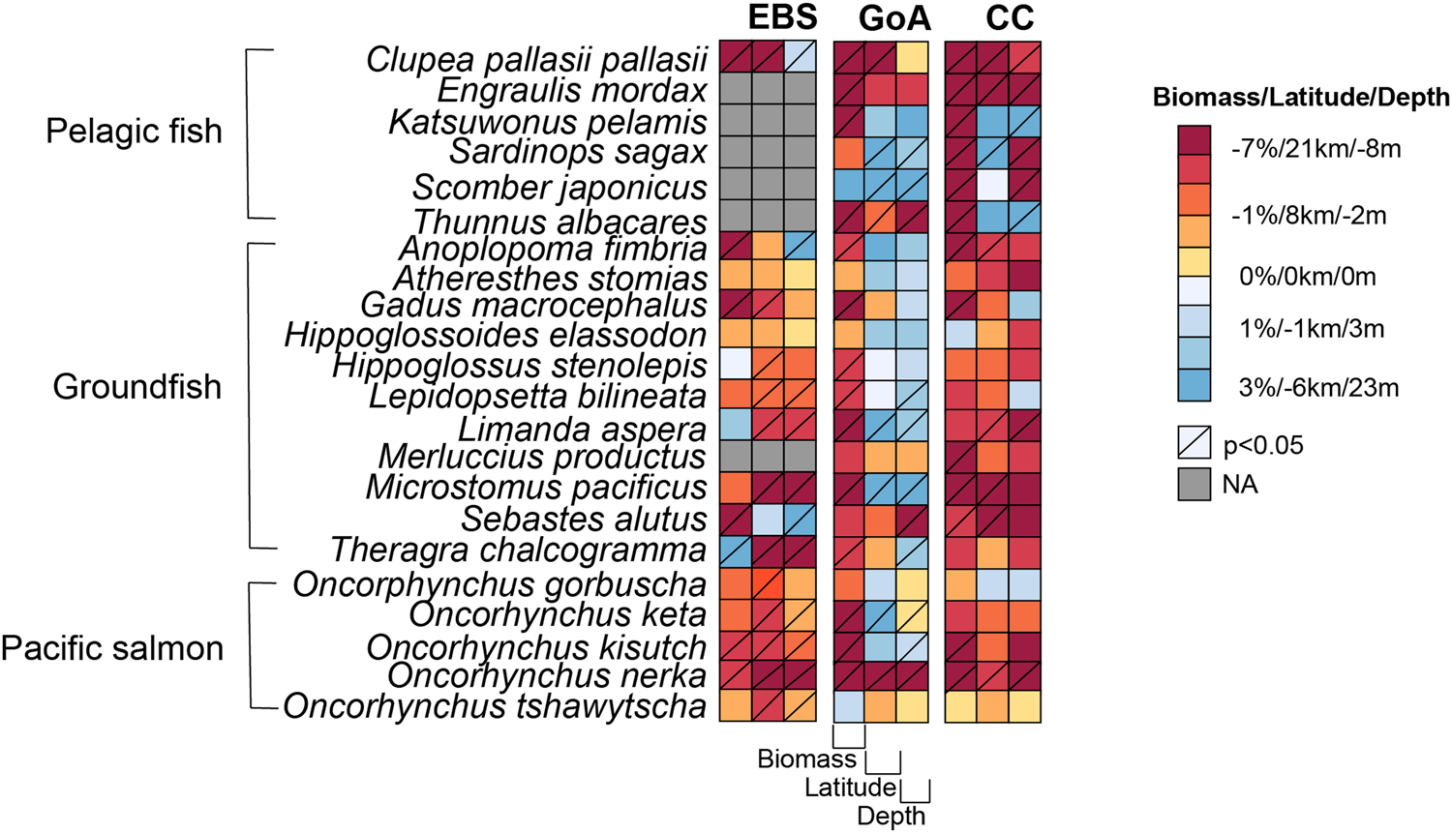
Projected mean changes in biomass, latitudinal and depth centroid of the 22 exploited marine fish stocks in the Eastern Bering Sea (EBS), Gulf of Alaska (GoA) and California Current (CC) during MHW years. NA - not available in catch record between 2006 and 2015.The different colour scales represent the projected changes in biomass, latitudinal centroid and depth centroid. Stripping of a cell represents changes at a significant level of below 0.05.

Amongst the 22 fishes, pelagic fish were projected to be most negatively impacted by MHWs, followed by Pacific salmon and groundfish (Fig. 4). Overall, almost all the studied pelagic fish showed significant decrease in biomass (more than 7%) under MHWs relative to the mean conditions, except Pacific sardine (*Sardinops sagax*) and Japanese mackerel (*Scomber japonicus*) that did not show significant changes in Gulf of Alaska. Amongst the five studied Pacific salmon species, biomass of sockeye salmon (*Oncorhynchus nerka*) decreased most substantially and most consistently across LMEs under MHWs, followed by coho salmon (*O. Kisutch)*. For groundfish, biomass of Pacific cod (*Gadus macrocephalus*), sablefish *(Anoplopoma fimbria*) and Pacific ocean perch (*Sebastes alutus*) were projected to decrease significantly under MHWs in all LMEs. Only Alaska pollock in the Eastern Bering Sea increased significantly in biomass under MHWs amongst all the 22 species and LMEs.

For MHW impacts on species’ biogeography, the distribution centroids of Pacific hering (*Clupea pallasii pallasii*) and sockeye salmon were projected to shift poleward in all three LMEs (Fig. 4). More fish stocks in Gulf of Alaska than in Eastern Bering Sea or California Current shifted significantly equatorward, or in variable directions amongst ensemble members under MHWs. Direction of shifts in depth centroid also often differed between LMEs for the same species. For example, Pacific Dover sole (*Microstomus pacificus*) was projected to shift poleward in Eastern Bering Sea and California Current, but equatorward in Gulf of Alaska and to deeper waters in Eastern Bering Sea, while shifted shallower in Gulf of Alaska (Fig. 4).

**Figure 5:**
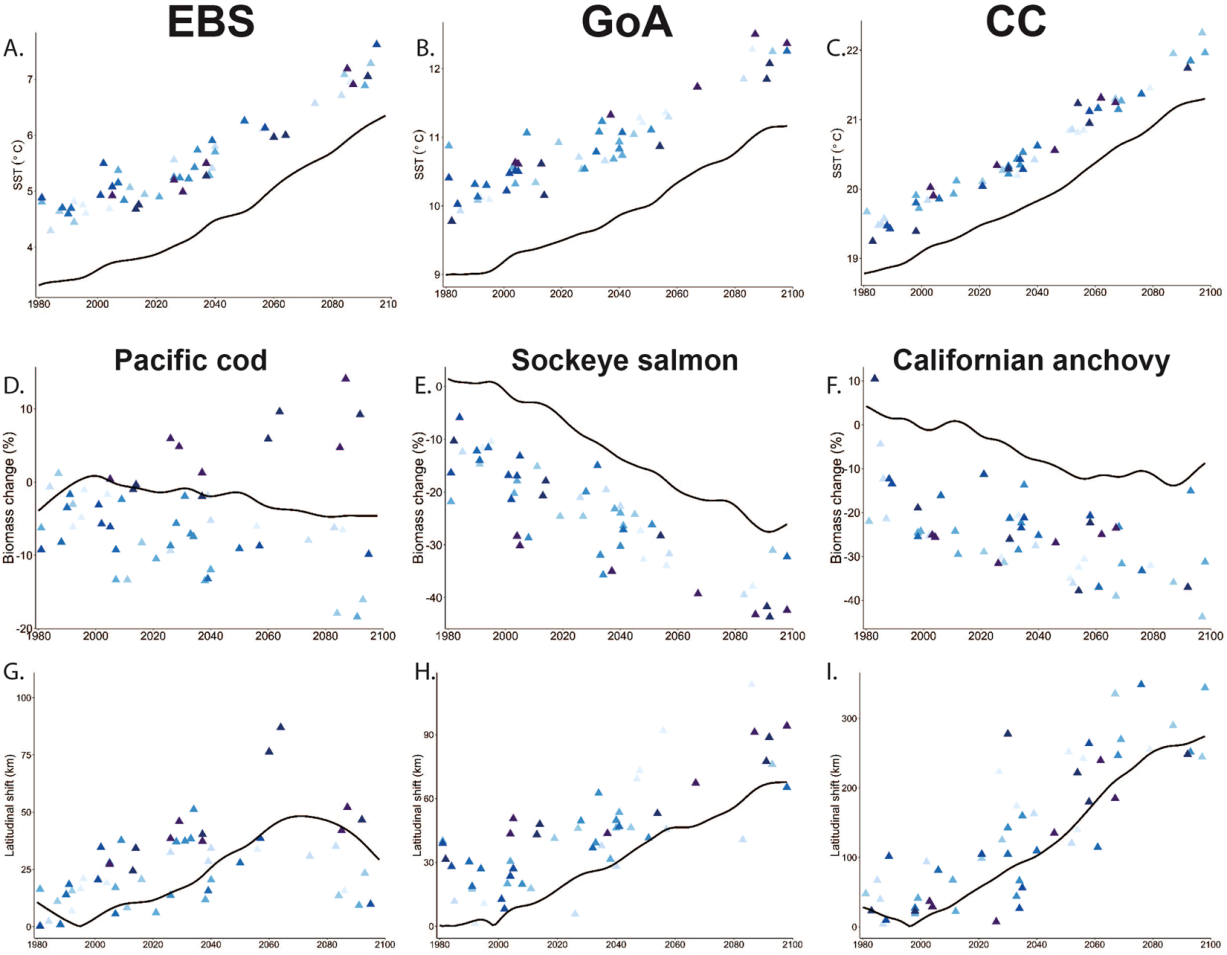
Projected time-series of changes in sea surface temperature (SST) (**A**–**C**), biomass (**D**–**F**) and latitudinal centroid (**G**–**I**): Pacific cod (*Gadus macrocephalus*) (**D**,**G**), sockeye salmon (*Oncorhynchus nerka*) (**E**,**H**) and Californian anchovy (*Engraulis mordax*) (**F**,**I**)) in the Eastern Bering Sea (EBS), Gulf of Alaska (GoA) and California Current (CC). The solid lines represent the average values across the 10 ensemble member simulations (smoothed with a cubic spline function); blue-colored triangles represent values during MHW years; the different intensity of blue color represents different ensemble member simulations (see Figs. S1–12 for results for all the large marine ecosystems and studied fish stocks).

We chose Pacific cod, sockeye salmon and Californian anchovy that were of particular interest to fisheries and coastal communities in the northeast Pacific region to highlight how MHWs will exacerbate impacts from long-term climate change (Fig. 5). Firstly, these species will experience ocean warming as a result of both the mean increase in SST under RCP8.5 as well MHWs (Fig. 5A–C). This will greatly exacerbate the warming hazards to these species. Secondly, biomass of these three fish stocks dropped approximately 5% for Pacific cod in Eastern Bering Sea to 30% for sockeye salmon in Gulf of Alaska and California anchovy in California Current during MHWs in addition to the decrease due to long-term mean changes under RCP8.5 (5%, 25% and 10% by 2100 relative to 2000; Fig. 5D–F). Similarly, shifts in biogeography, as indicated by the latitudinal centroids of the three selected species (Fig. 5G–I), added to the effects of the shifts due to changes in mean ocean conditions by as much as 100 km poleward during MHWs (e.g., California anchovy in California Current). As such, biomass decrease and biogeographic shifts during MHWs early in the 21st century were projected to be at a similar level as the decadal-scale average changes by around the 2050 s. This also means that MWHs will exert large impact ‘shocks’ while fish stocks are already impacted by long-term mean climate change. For example, with both MHWs and changes in mean conditions, biomass of sockeye salmon was projected to drop by more than 40% by 2100 relative to 2000 under RCP8.5.

## Discussion

Our findings provide theoretical support to the empirical observations from scientific surveys and anecdotal accounts from fishers that fisheries important fish stocks such as Pacific cod and sockeye salmon had been impacted by the 2013–2015 northeast Pacific MHW^19^. In addition, we offer new insights into the combined impacts of MHWs and long-term climate change on the species distribution in the northeast Pacific. Specifically, we show that MHWs can more than double the magnitude of the impacts on fish stocks by 2050 due to long-term climate change. Previous vulnerability and impact assessments have therefore greatly underestimated the risk to future fish stocks and fisheries in the northeast Pacific under climate change.

Some fish stocks had already showed changes in biogeography during the recent Blob that are similar to the MHW impacts projected in this study. In Gulf of Alaska, analysis using data collected from scientific surveys showed that some groundfish species such as Pacific cod had shifted their distributions to deeper waters during anomalous warm temperature^8,9^. However, the directions of biogeographic shifts varied between species and across their life stages. Such shifts also differed between sub-regions due to different oceanographic conditions and bathymetric profiles^8^. These oceanographic and biological complexities could contribute to the large variability of our projected biogeographic shifts for groundfish between ensemble members.

Although shifts in biogeography associated with the Blob are more widely reported in literature^19^, our results show that biomass decreases are more consistent in response to MHWs in the northeast Pacific relative to biogeographic shifts. Therefore, biomass of fish stocks may be a better impact indicator in detecting and assessing the impacts of MHWs as part of ecosystem-based management. However, as the magnitude of the projected biomass decrease and biogeographic shifts varied between species in our study, different sets of impact indicators that are species-specific can be used to more efficiently monitor and assess the impacts of MHWs.

The characteristic of the MHW impacts will result in a different set of challenges for management and conservation of living marine resources than those associated with the long-term mean change in climate. The rate of changes in biomass, potential catches and biogeography of fish stocks are much higher under MHWs than under long-term climate change. For example, in the California Current, Pacific sardine and California anchovy population are observed to show alternations of their abundance that are partly driven by changes in oceanographic regimes in the Pacific Ocean^38,39^. Particularly, warm regimes tend to favor sardine’s recruitment and abundance while cool regimes favor anchovy. Thus, under decade-scale mean ocean warming, sardine was projected to increase in biomass while the opposite was projected for anchovy in the California Current. In contrast, poleward range expansion of sardine and anchovy was projected to result in long-term increase in their abundance in the Gulf of Alaska. However, the projected short-term rapid warming under MHWs pushed environmental temperature beyond those preferred by both sardine and anchovy, leading to a drop in their biomasses in both the California Current and Gulf of Alaska. Moreover, satellite data and model simulations suggest that MHWs are linked to and can be exacerbated by, multi-annual climate variability such as El Niño Southern Oscillation (ENSO), resulting in the particularly large and persistent biological impacts in the Northeast Pacific region from the Blob^13^,^19^,^40^. In any case, these complex biological responses of sardine and anchovy that inter-mixed between the effects of MHWs and decadal-scale warming therefore demand more rapid and short-term governance and adaptation responses such as alteration of fishing quota, shifts in fishing ground and targeted species^41^. The challenges from MHWs impact will thus put ‘double strains’ on sustainable management of living marine resources under climate change, pointing to the need for future research into the development of more robust adaptation and governance responses^42,43^. Previous studies have shown that global warming substantially increases the risks of MHWs to occur^14^. Our study additionally suggests that MHWs can strongly exacerbate the impact of decadal-scale mean ocean warming on fish stocks. A reduction of anthropogenic greenhouse gas emissions - the fundamental driver of global warming^44^ – is therefore needed to limit the impacts of MHWs on fish stocks and fisheries^5^.

Even though we consider the projected pattern of MHW impacts on fish stocks and the implications for understanding future risks on fisheries and their governance under climate change as robust, a number of caveats needs to be discussed. The global Earth system model used in this study (i.e. GFDL ESM2M) is able to adequately simulate mean states and trends in different marine heatwave metrics over the satellite 1982–2016 period^14^. However, the horizontal resolution (about 1°) of the ocean component of the Earth system model is too coarse to accurately represent some of the oceanographic dynamics in coastal and shelf seas such as upwelling or mesoscale eddy activity e.g.^45^. In addition, some of the biogeochemical processes in the high latitudes associated with sea ice are also not well resolved. The simulated net primary productivity in GFDL ESM2M, in particular, is highly uncertain^46^, especially in regions with sea ice^47^, because nutrient inputs during sea ice melt^48^ or through rivers^49^ are not included. The fish stock model assumes that historical species’ biogeography reflects their environmental niches^50^. Variations in the projected pathways of changes in biomass and biogeography of species in this study were partly caused by the differences in species’ temperature preferences calculated from different Earth system model ensemble members e.g., the increase in biomass under MHWs for Pacific cod in one of the ensemble members (dark purple diamonds in Fig. 5G). The fish stock model also did not account for interspecific interactions or evolutionary adaptation to epigenetic responses to environmental changes^51^. For instance, we projected a positive impact of MHWs on Alaskan pollock (*T. chalcogramma*) in Eastern Bering Sea. However, previous studies have suggested that anomalous warm temperature affects the availability of preferred nutritious prey that reduced the survivorship and recruitment of pollock in the Bering Sea^19^. Moreover, we only examined climate projections following the ‘no mitigation’ high greenhouse gas emissions scenario (RCP8.5) and including an ‘idealized’ fishing scenario i.e., assuming all fishing is at level to achieve maximum sustainable yield of each fish stock. The effects of scenario uncertainties associated with different greenhouse gas emission and pathways of fishing effort and their management on the impacts of MHWs on marine ecosystems need to be explored further. Future research can build on the foundation laid by this study to incrementally address these uncertainties^52^. For example, the number of ensemble members, Earth system models and fish models may be increased to explore a wider range of model uncertainties. The analysis can also be repeated using high resolution Earth system models, and fish models with trophic interactions and/or eco-evolutionary dynamics.

Overall, this study underscores the importance of considering MHWs in assessing climate risks and impacts. Previous risk and impact assessment that focused on the effects of long-term changes in mean conditions under climate change may have largely underestimated climate risks on fish stocks and fisheries. Moreover, the rapid rate of change and the prevalence of impacts across fisheries important fish stocks in the northeast Pacific point to the need to examine whether climate adaptation, designed mostly for dealing with long-term mean changes, would be sufficient to reduce the additional climate risks from MHWs. Without appropriate mitigation and adaptation measures, MHWs may pose additional risks on the long-term viability of marine species and the sustainability of their fisheries, and the associated benefits to dependent human communities such as food, economic benefits and livelihoods^11^. Our results also provide a foundation for further modelling efforts and analysis to build on and systematically explore different dimensions of uncertainties.

## Methods

### Earth system model

We analyzed projected changes in annual mean SST in the northeast Pacific Ocean using a 10-member ensemble simulation of the Earth system model version 2 developed at the Geophysical Fluid Dynamics Laboratory (GFDL ESM2M^33–35^). The GFDL ESM2M is a fully coupled carbon cycle-climate model that consists of an ocean, atmosphere, sea ice, and land model, and includes land and ocean biogeochemistry. The nominal horizontal resolution of the ocean component is about 1° latitude × 1° longitude with 50 vertical levels^53^.

Each ensemble simulation is run over the 1950–2100 period under the same external forcing of historical changes before 2005 and Representative Concentration Pathway 8.5 (RCP8.5) afterwards. The RCP8.5 is a high greenhouse gas emission scenario^54^ that leads to a global atmospheric surface warming in ESM2M of 3.2 °C by 2081–2100 relative to preindustrial. All 10 ensemble members are run under the same external radiative forcing scenario, but are started from different initial conditions in January 1st of 1950. Spread in the ensemble members is generated by slightly perturbing the initial state of the Earth system at the start of each simulation. These initial perturbations cause each ensemble member to have a unique atmosphere and ocean state at each point in time, i.e. a different state of internal variability. As a specific example, the real ocean experienced an El Niño in 1997–1998. In the model, ensembles may have had a La Niña, El Niño or been neutral at this time.

### Dynamic bioclimate envelope model

We simulated changes in abundance and distributions of 22 exploited fish stocks that are highly important to fisheries in the northeast Pacific region using the dynamic bioclimate envelope model (DBEM)^36,37^. The DBEM is a spatially-explicit biomass dynamic model. It is driven by changes in ocean conditions that are obtained from the Earth system model simulations described above. Variables of ocean conditions include temperature, dissolved oxygen concentration, salinity, sea ice extent, surface advection and net primary production. Variables for surface and bottom were applied to model pelagic and demersal species, respectively. The DBEM model simulates changes in annual average biomass and catch potential of marine fishes and invertebrates on a 0.5^o^ latitude × 0.5^o^ longitude grid of the world ocean. Movement of adults and pelagic larvae is calculated by sets of advection and diffusion equations with diffusion rates vary according to gradients of environmental suitability for each modelled species and ocean currents^36^. Fishing mortality (F) was set at the level to achieve maximum sustainable yield (MSY). The projected annual catch for each species is hereafter termed maximum catch potential.

### Identifying MHWs in the northeast Pacific

To identify MHWs, we calculated anomalies between the SST simulated by each of the individual 10 ensemble members and the ensemble-averaged SST (Fig. 2). First, for each ensemble member simulation, we calculated the annual average SST in each of the three LMEs within the northeast Pacific region (as defined in Fig. 1) from 1950 to 2100 (blue line in Fig. 2a). Second, for each year, we calculated the average SST across the temperatures simulated from the 10 ensemble members; i.e. the ensemble-averaged SST (black line in Fig. 2a). Third, we applied a cubic spline (using the R function “smooth.spline” with smoothing parameter = 0.6) to the ensemble mean SST to further minimize the contribution of changes in temperature due to internal variability (orange line in Fig. 2a). Thus, the resulting SST series show the long-term changes in mean conditions only. For each LME in the northeast Pacific region, we identified MHWs as the positive temperature anomalies that were above the 95th percentile of temperature anomalies from 1950 to 2100 (red line in Fig. 2a). For every MHW identified from each ensemble member, we characterized its magnitude (SST anomalies relative to the smoothed ensemble mean values) and occurrence year.

### Modelling ecological responses to MHWs

We included a total of 22 fish species that were reported in the fisheries statistics in the northeast Pacific region (www.seaaroundus.org). These species were important to fisheries in this region as they contributed up to 80% of the total observed catches from 2006 to 2015 (www.seaaroundus.org). We calculated four impact indicators to examine the ecological responses of fish stocks and their implications for fisheries during a MHW. These indicators are: (1) total biomass, (2) latitudinal centroid (average of the coordinates of grid cell weighted by the species’ biomass), (3) depth centroid (average of bathymetry of grid cell weighted by the species’ biomass), and (4) maximum catch potential (catch at F = F_MSY_). We used outputs from DBEM to calculate these indicators for each LME. Since the projected relative changes in biomass and maximum catch potential are similar, we presented simulation outputs for changes in biomass only.

For each of the four impact indicators, we calculated the annual anomalies with procedures similar to those applied to SST (Fig. 2). Firstly, we applied DBEM to simulate changes in spatial distribution of biomass and catches from 1950 to 2100 under changes in ocean conditions projected from each of the 10 Earth system model ensemble members (blue line in Fig. 2b). Secondly, for each year, we calculated the average values simulated from the 10 ensemble members (black line in Fig. 2b) and smoothed the averaged series with a cubic spline filter (orange line in Fig. 2b). We then calculated the annual anomalies of each impact indicator from the difference between each ensemble member simulation and the detrended series. Finally, we recorded the ensemble member-specific annual indicator anomalies in the year when the temperature anomalies had been characterized as MHWs (red bars in Fig. 2b). We focused on analyzing the simulated impact indicators from 1981 to 2100 to ensure that the detected signals are not due to model initialization during the early period of the simulation.

We tested the statistical significance of the effects of the occurrences of MHWs on the ecological impact indicators for exploited fish stocks in the northeast Pacific region using the glm function in R, with the occurrences of MHWs or non-MHW year as factor. The datasets for the information for accessing the projected temperature changes, MHWs and impacts on fish stocks are provided in the SI.

## Supplementary information

Supplementary Information.
