# Supplementary material for: Marine heatwaves exacerbate climate change impacts for fisheries in the northeast Pacific
Source: Sci Rep. 2020 Apr 21;10:6678. doi: 10.1038/s41598-020-63650-z (PMC7174322; doi:10.1038/s41598-020-63650-z)
Supplement: Supplementary file 1 — Supplementary Information. [file 41598_2020_63650_MOESM1_ESM.docx]

Marine heatwaves exacerbate climate change impacts for fisheries in the northeast Pacific

Short title: Marine heatwaves exacerbate impacts on fisheries

**Authors**

William W. L. Cheung^1,*^, Thomas L. Frölicher^2,3^ †

**Affiliations**

1. * Changing Ocean Research Unit, Institute for the Oceans and Fisheries, The University of British Columbia, Vancouver, B.C., Canada, V6T 1Z4. Correspondence to email. w.cheung@oceans.ubc.ca
2. Climate and Environmental Physics, Physics Institute, University of Bern, Switzerland
3. Oeschger Centre for Climate Change Research, University of Bern, Switzerland

†W. Cheung conducted the study as a visiting scholar at the Oeschger Centre for Climate Change Research and the Climate and Environmental Physics Division of the Physics Institute of the University of Bern.

## **Supplementary information**


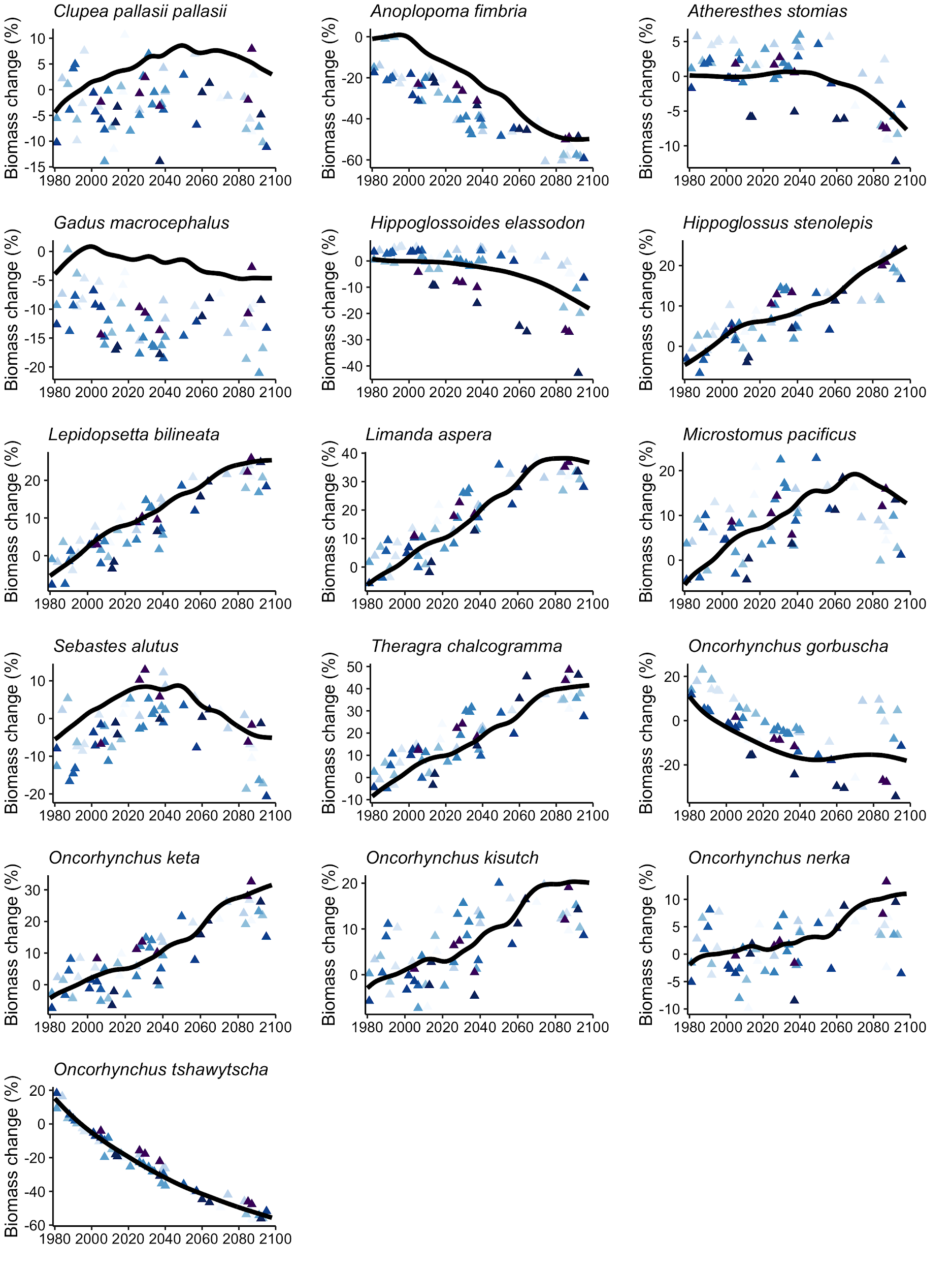


**Fig. S1. Projected time-series of biomass of the studied fish stocks in the Eastern Bering Sea.** The solid lines represent the average values across the 10 ensemble member simulations (smoothed with a cubic spline function); blue-coloured triangles represent values during MHW years; the different intensity of blue colour represents different ensemble member simulations.


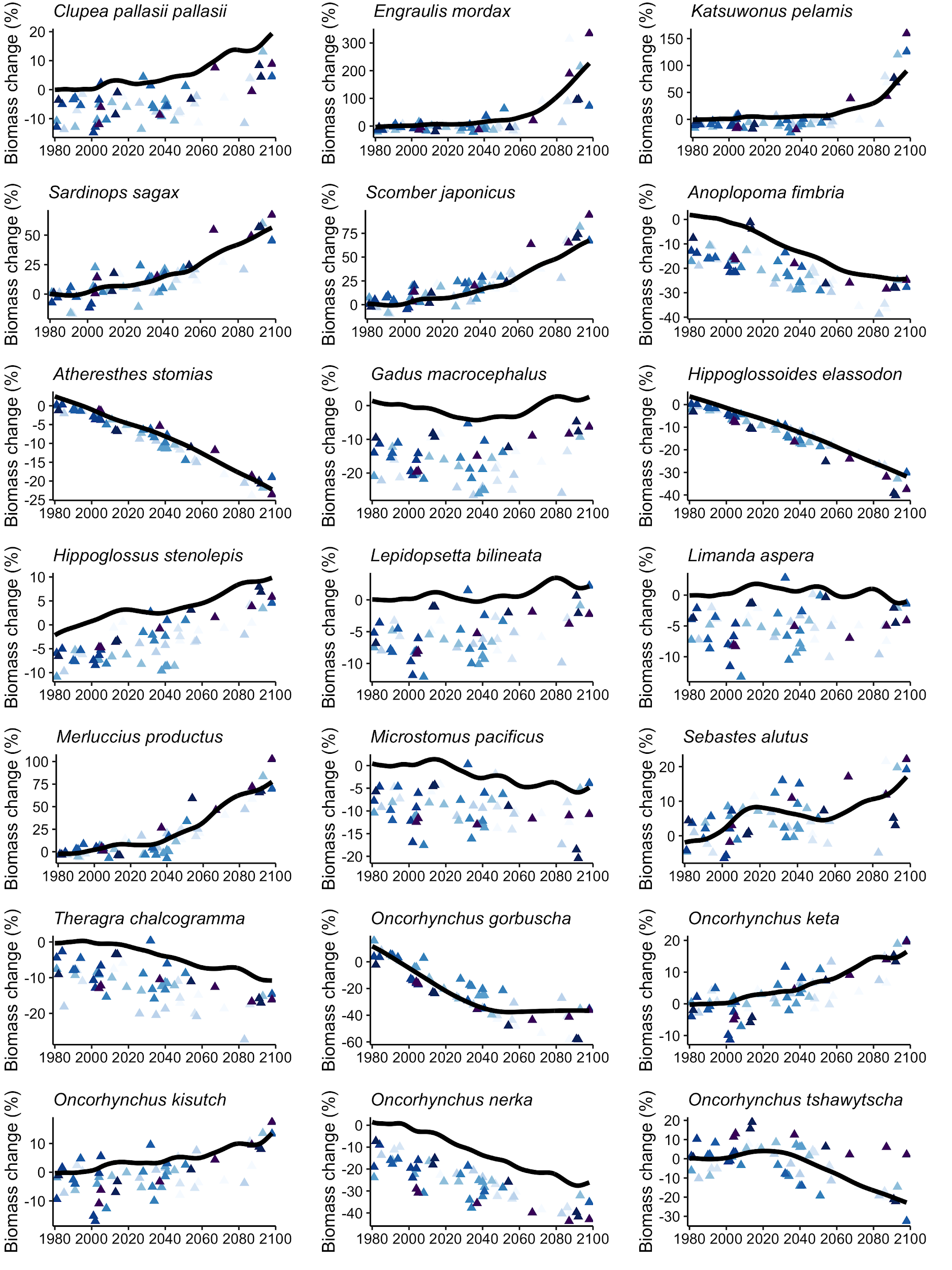


**Fig. S2. Projected time-series of biomass of the studied fish stocks in the Gulf of Alaska.** The solid lines represent the average values across the 10 ensemble member simulations (smoothed with a cubic spline function); blue-coloured triangles represent values during MHW years; the different intensity of blue colour represents different ensemble member simulations.


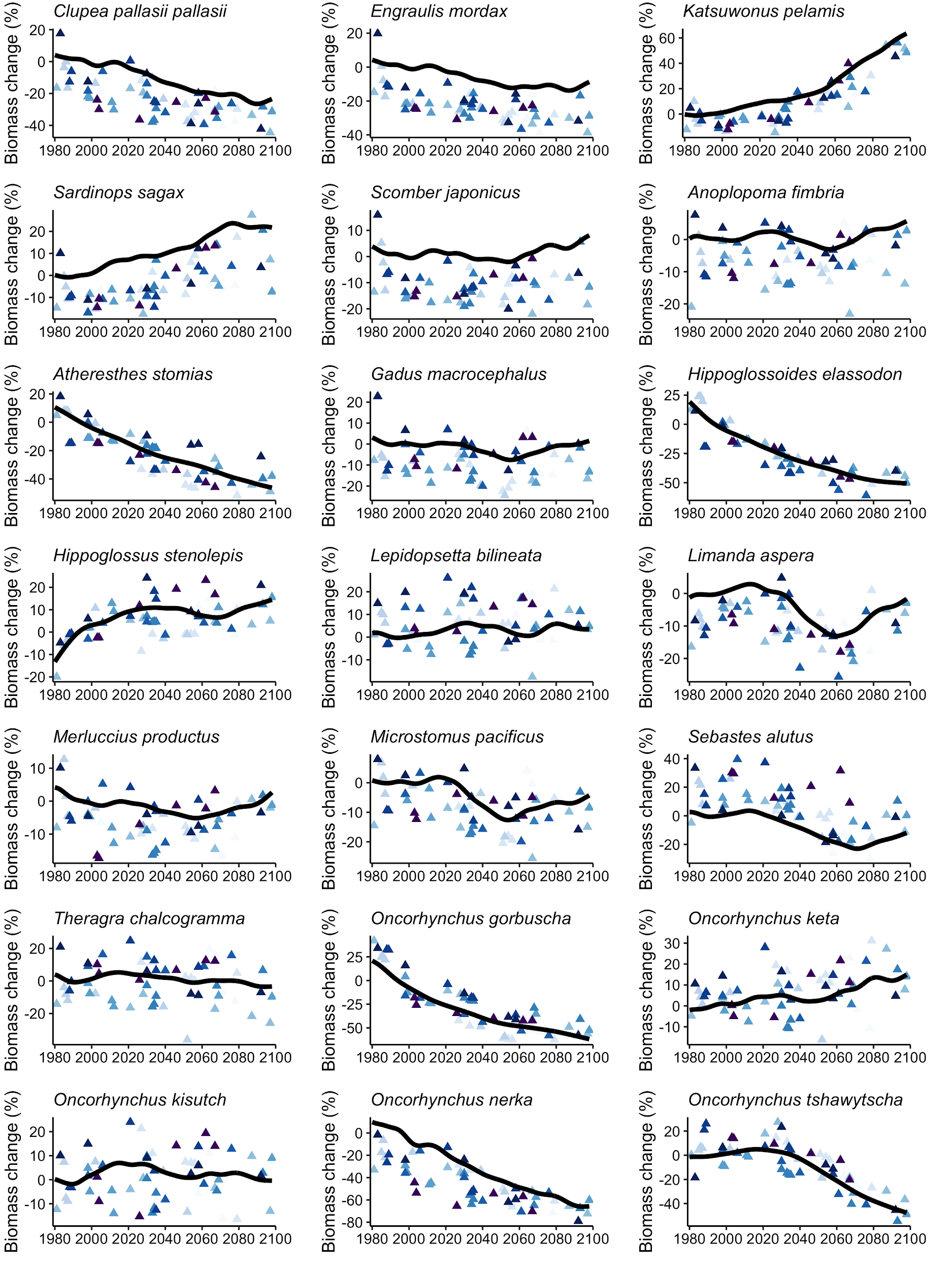


**Fig. S3. Projected time-series of biomass of the studied fish stocks in the California Current.** The solid lines represent the average values across the 10 ensemble member simulations (smoothed with a cubic spline function); blue-coloured triangles represent values during MHW years; the different intensity of blue colour represents different ensemble member simulations.


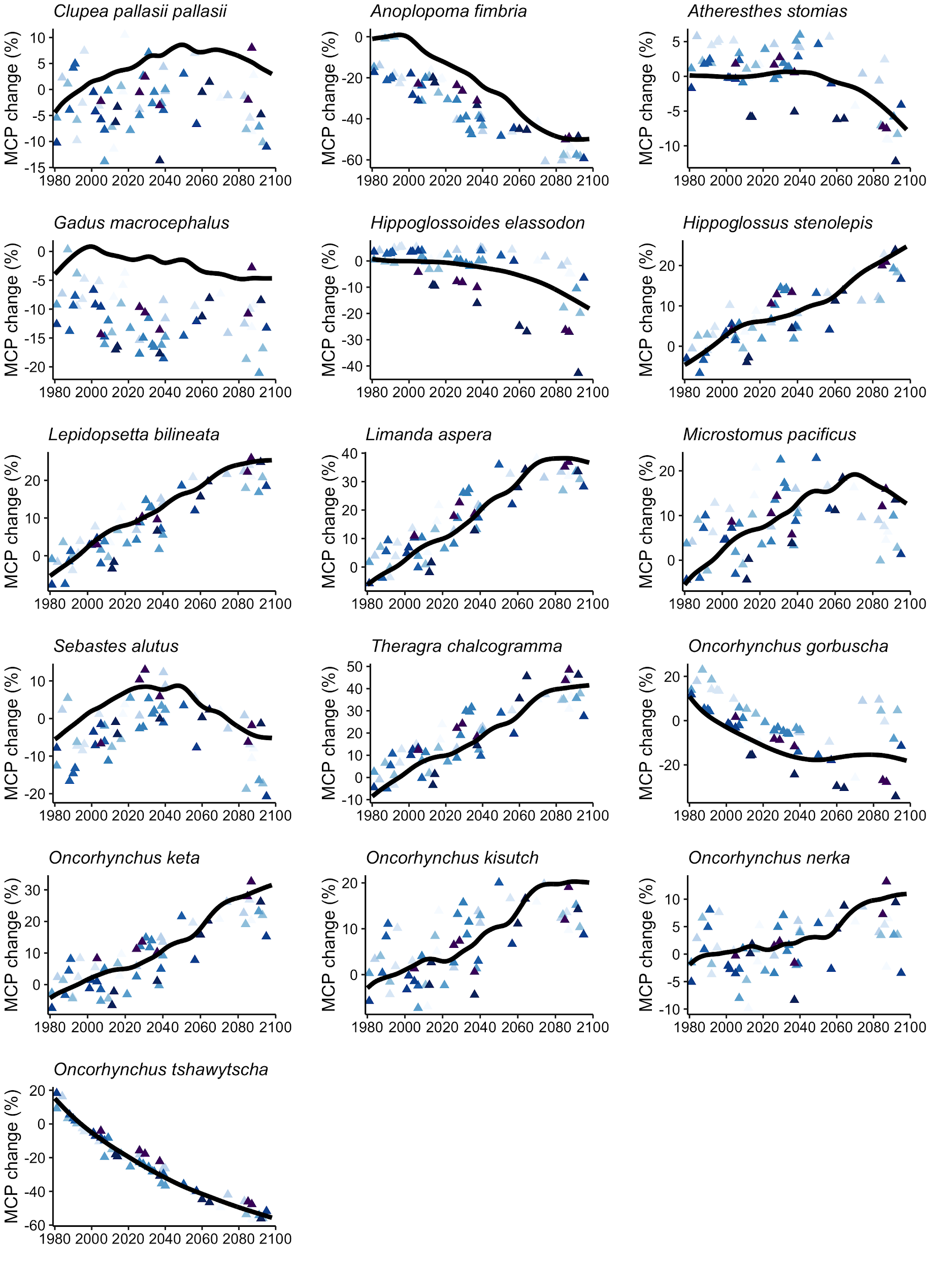


**Fig. S4. Projected time-series of maximum catch potential (MCP) of the studied fish stocks in the Eastern Bering Sea.** The solid lines represent the average values across the 10 ensemble member simulations (smoothed with a cubic spline function); blue-coloured triangles represent values during MHW years; the different intensity of blue colour represents different ensemble member simulations.


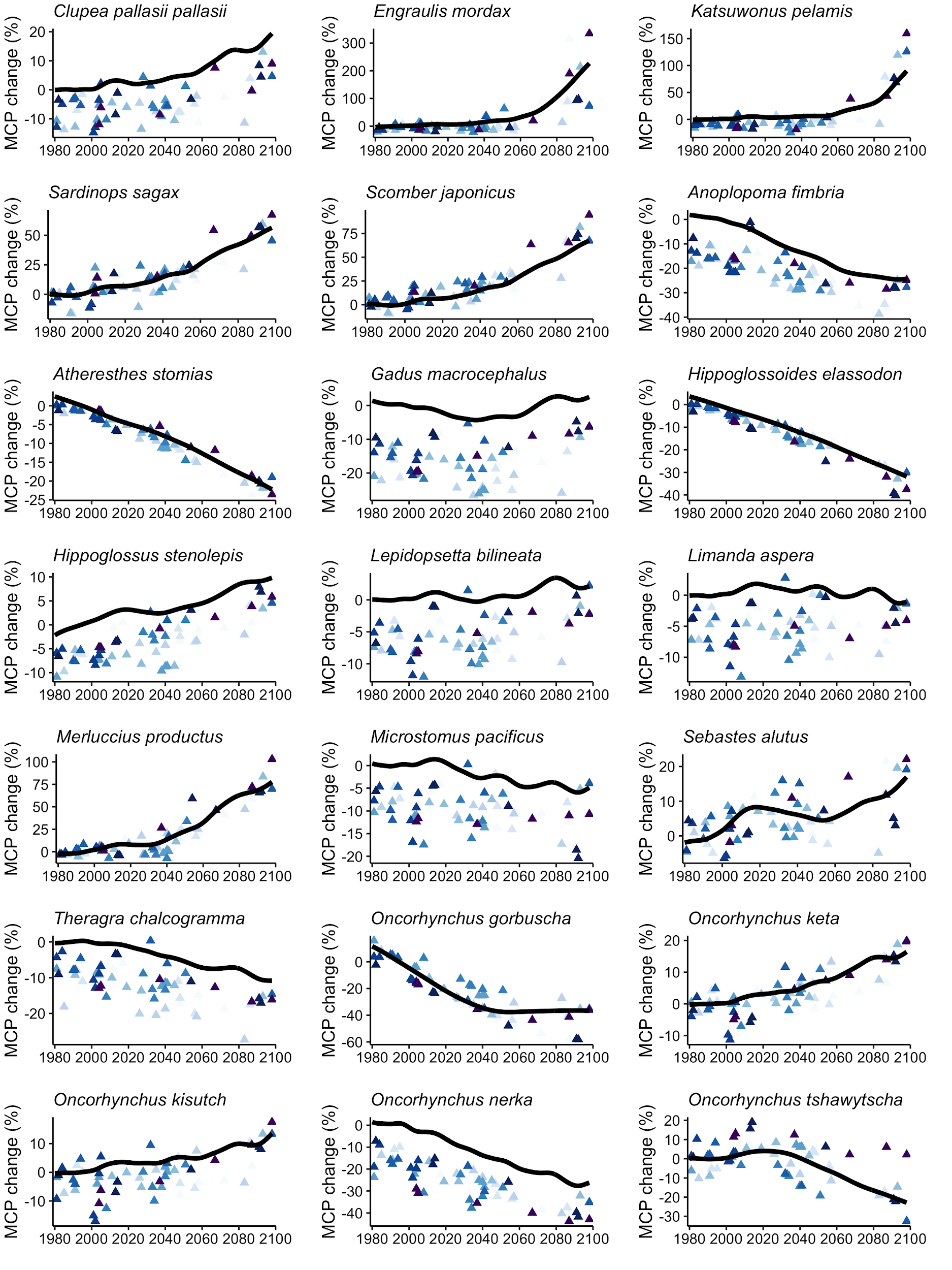


**Fig. S5. Projected time-series of maximum catch potential (MCP) of the studied fish stocks in the Gulf of Alaska.** The solid lines represent the average values across the 10 ensemble member simulations (smoothed with a cubic spline function); blue-coloured triangles represent values during MHW years; the different intensity of blue colour represents different ensemble member simulations.


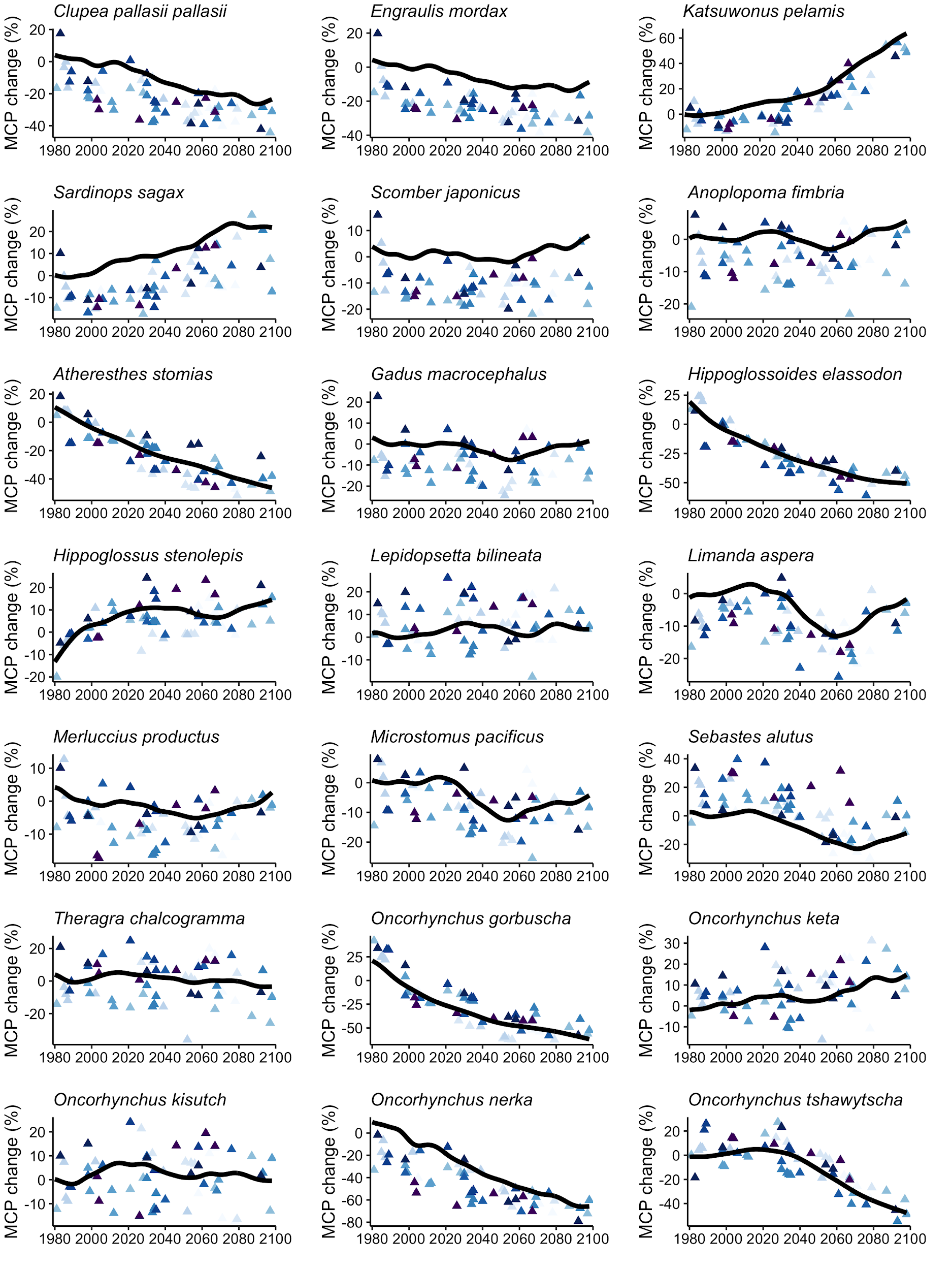


**Fig. S6. Projected time-series of maximum catch potential (MCP) of the studied fish stocks in the California Current.** The solid lines represent the average values across the 10 ensemble member simulations (smoothed with a cubic spline function); blue-coloured triangles represent values during MHW years; the different intensity of blue colour represents different ensemble member simulations.


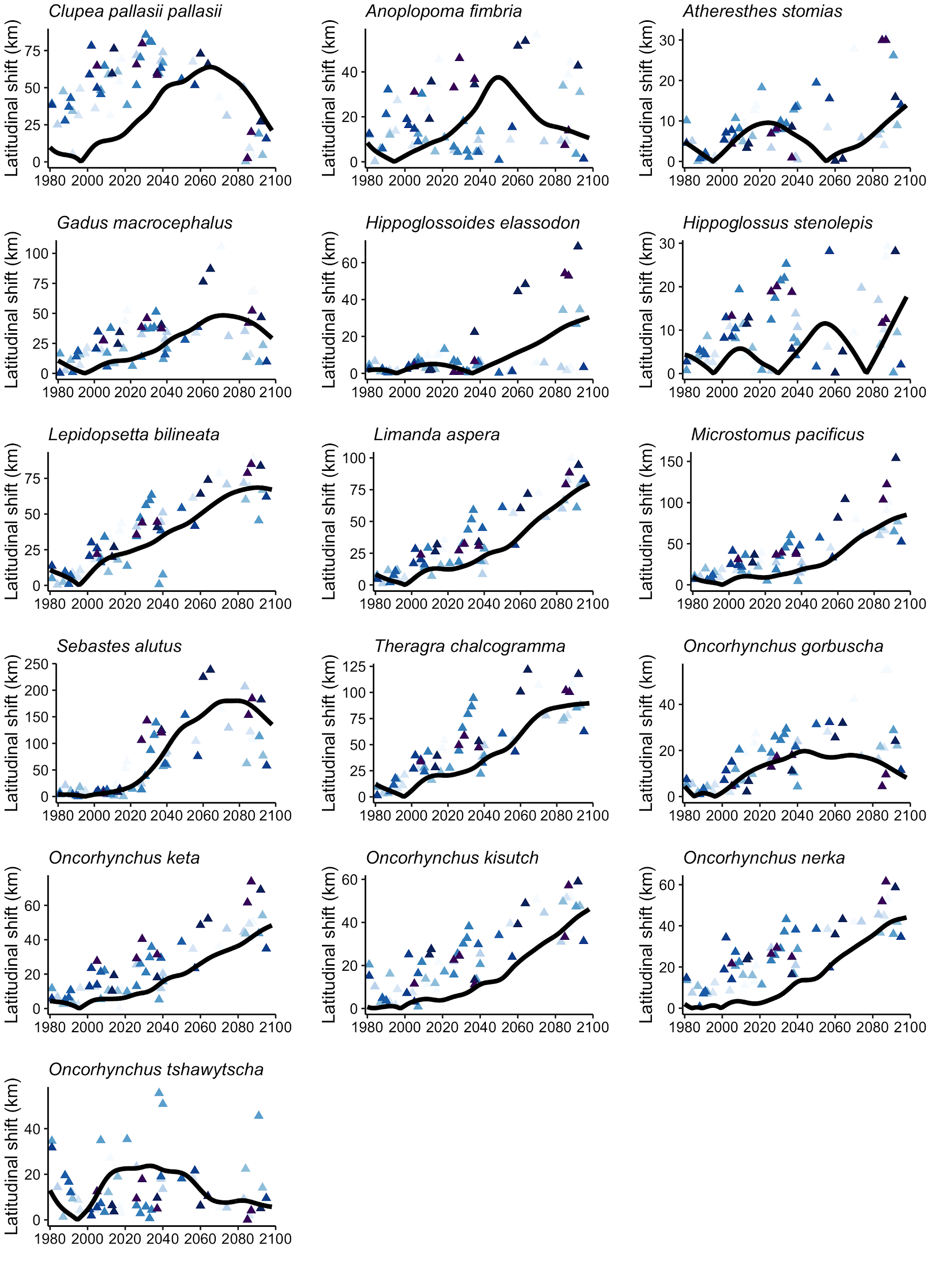


**Fig. S7. Projected time-series of latitudinal centroid of the studied fish stocks in the Eastern Bering Sea.** The solid lines represent the average values across the 10 ensemble member simulations (smoothed with a cubic spline function); blue-coloured triangles represent values during MHW years; the different intensity of blue colour represents different ensemble member simulations.


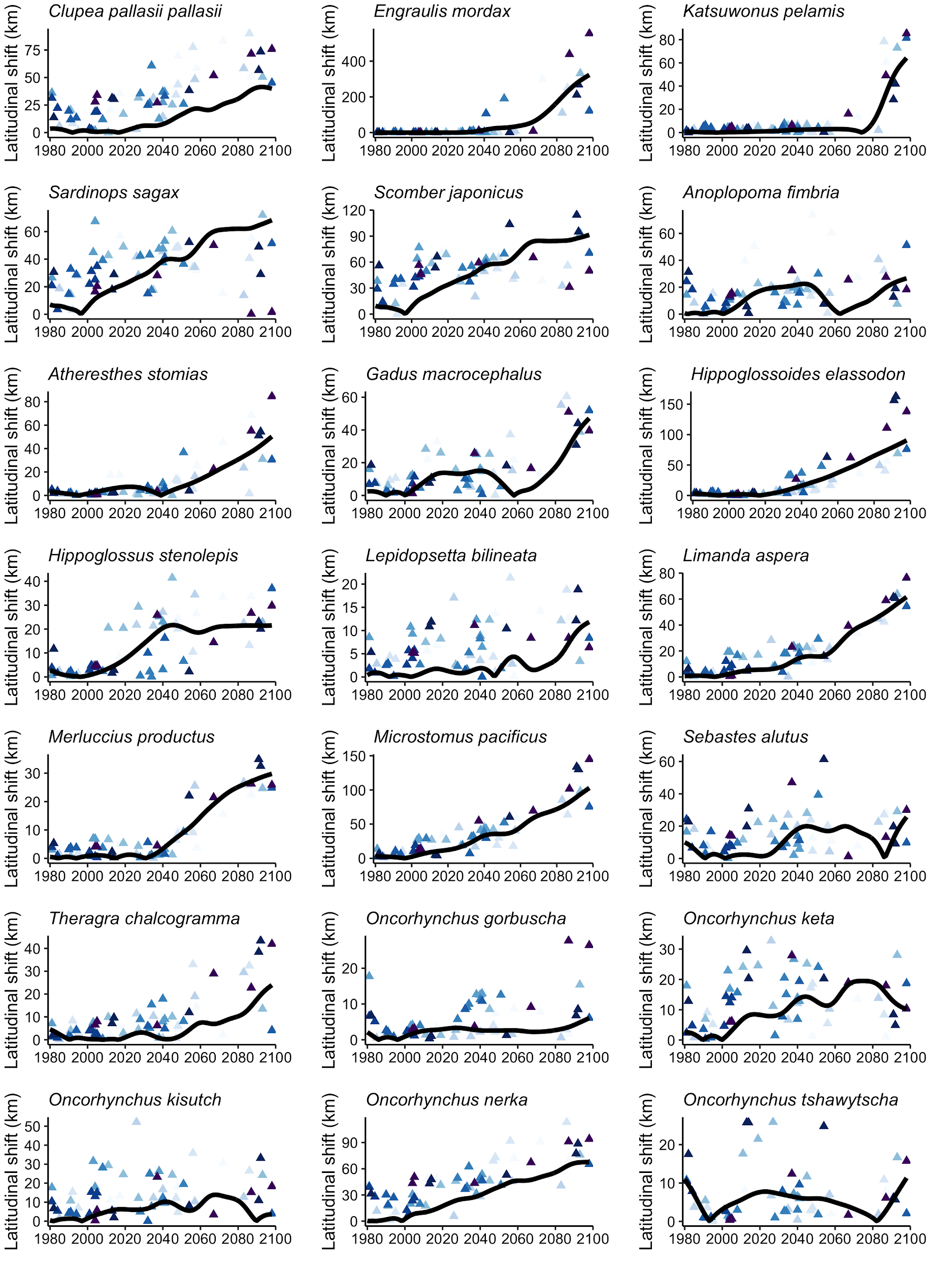


**Fig. S8. Projected time-series of latitudinal centroid of the studied fish stocks in the Gulf of Alaska.** The solid lines represent the average values across the 10 ensemble member simulations (smoothed with a cubic spline function); blue-coloured triangles represent values during MHW years; the different intensity of blue colour represents different ensemble member simulations.


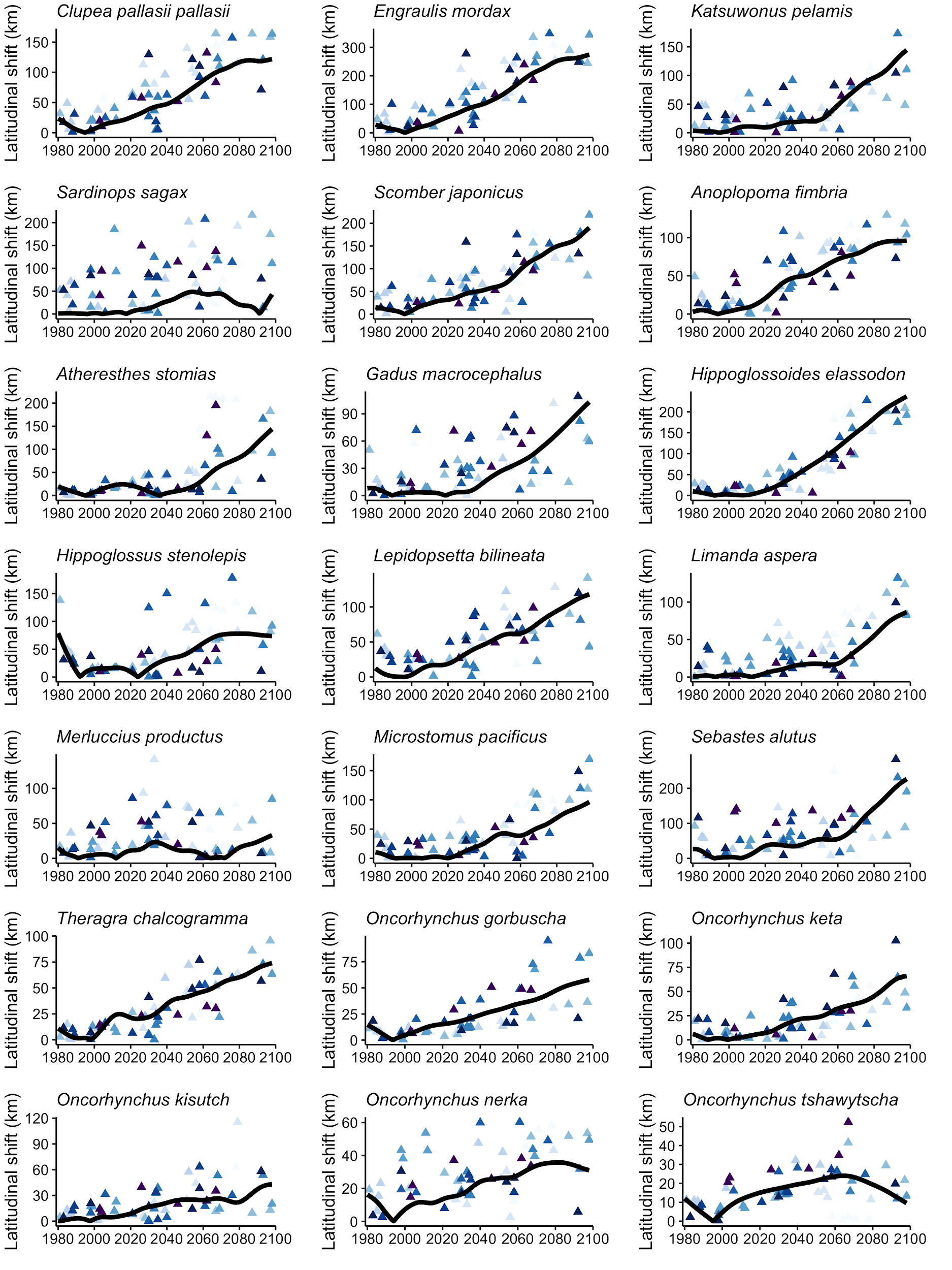


**Fig. S9. Projected time-series of latitudinal centroid of the studied fish stocks in the California Current.** The solid lines represent the average values across the 10 ensemble member simulations (smoothed with a cubic spline function); blue-coloured triangles represent values during MHW years; the different intensity of blue colour represents different ensemble member simulations.


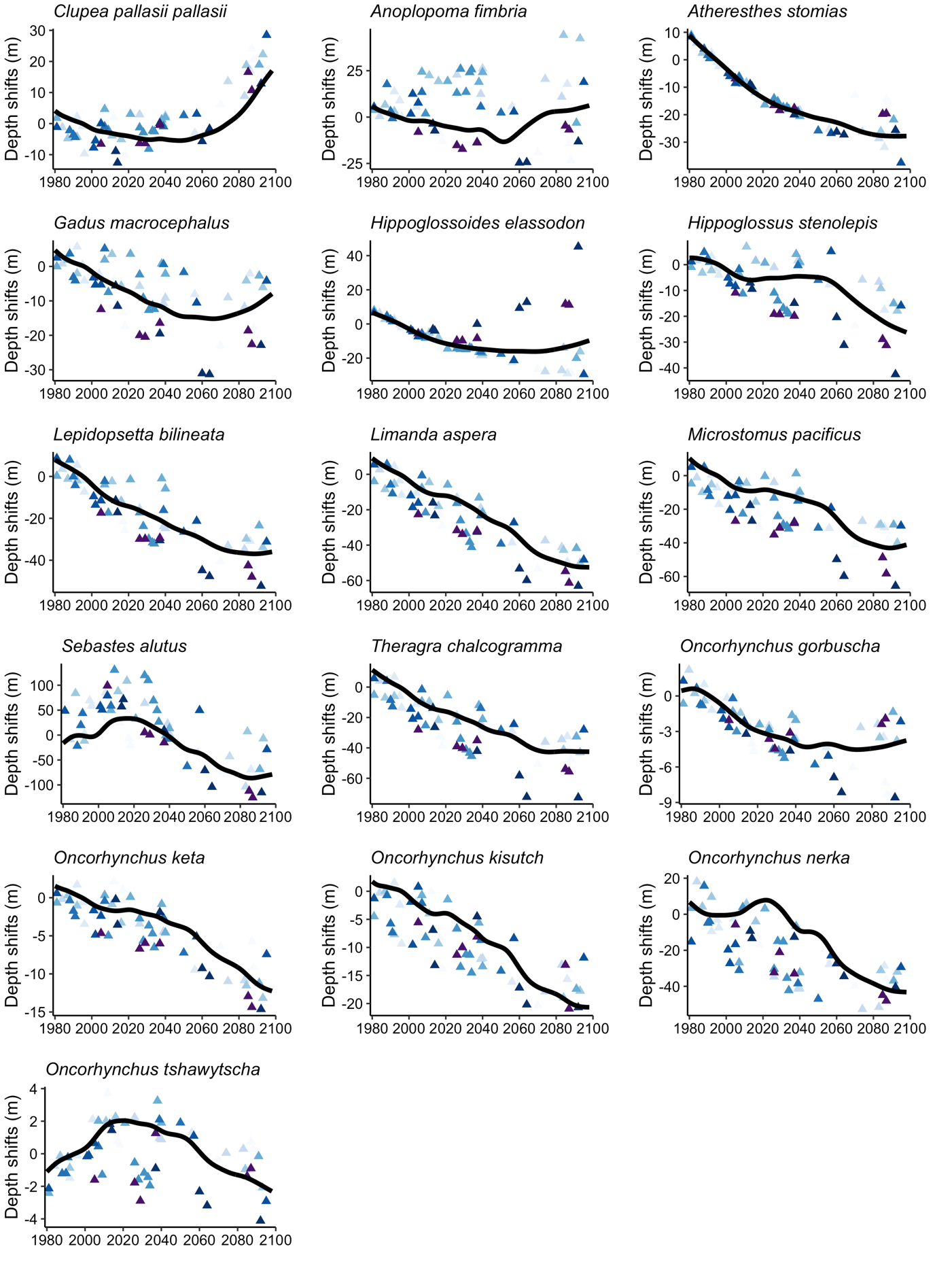


**Fig. S10. Projected time-series of depth centroid of the studied fish stocks in the Eastern Bering Sea.** Positive values indicate shifts to shallower waters. The solid lines represent the average values across the 10 ensemble member simulations (smoothed with a cubic spline function); blue-coloured triangles represent values during MHW years; the different intensity of blue colour represents different ensemble member simulations.


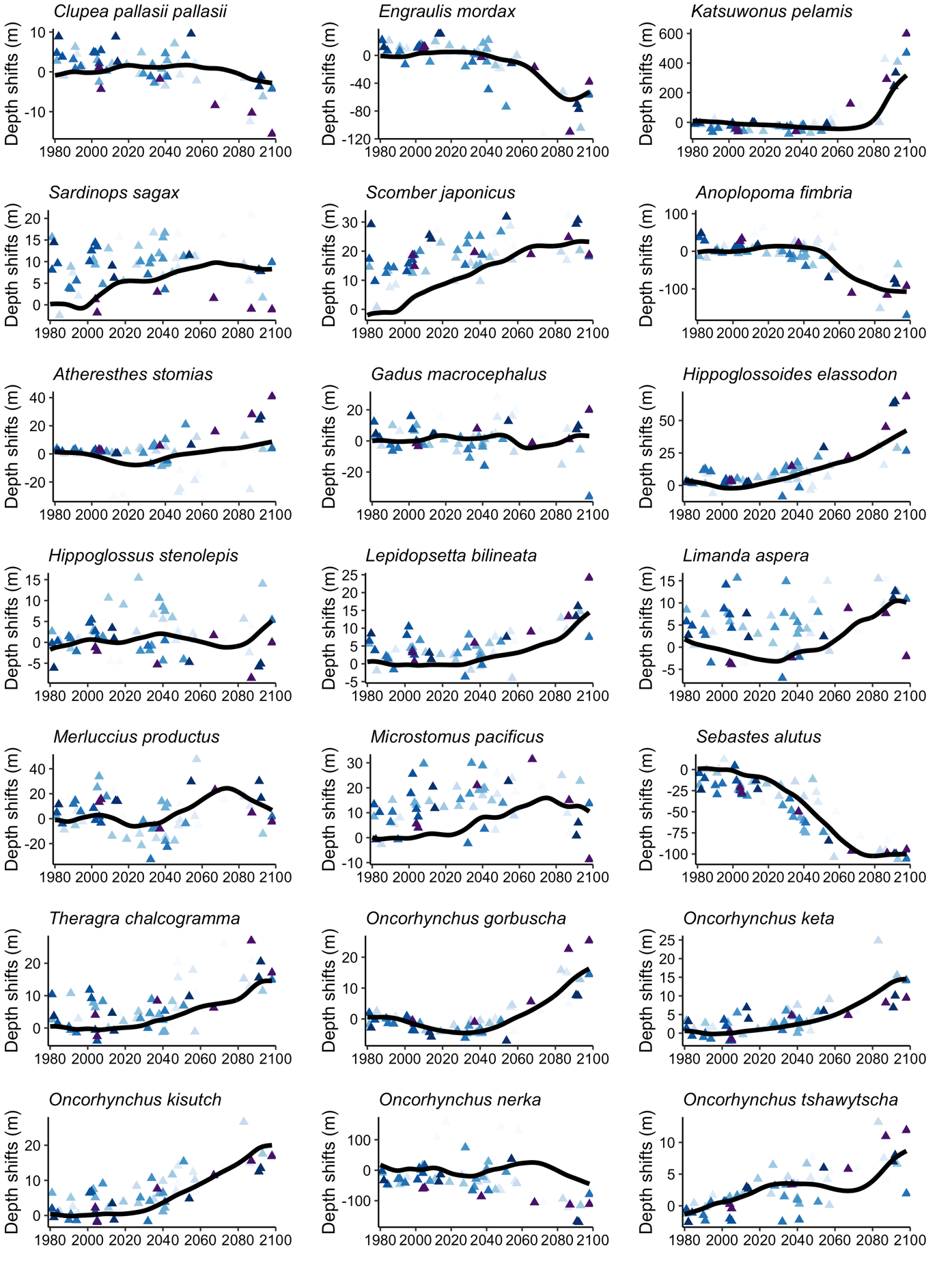


**Fig. S11. Projected time-series of depth centroid of the studied fish stocks in the Gulf of Alaska.** Positive values indicate shifts to shallower waters. The solid lines represent the average values across the 10 ensemble member simulations (smoothed with a cubic spline function); blue-coloured triangles represent values during MHW years; the different intensity of blue colour represents different ensemble member simulations.


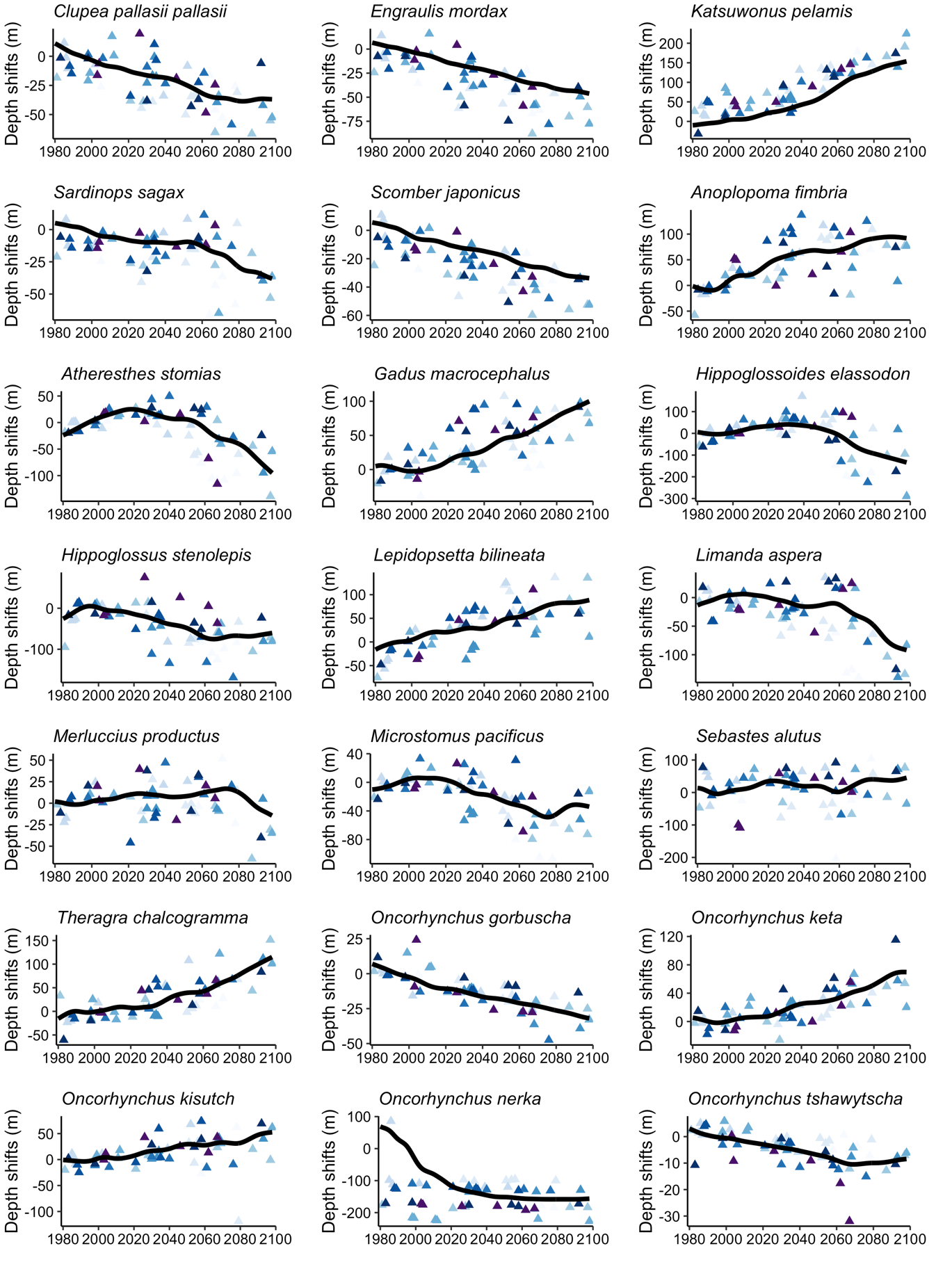


**Fig. S12. Projected time-series of depth centroid of the studied fish stocks in the California Current.** Positive values indicate shifts to shallower waters. The solid lines represent the average values across the 10 ensemble member simulations (smoothed with a cubic spline function); blue-coloured triangles represent values during MHW years; the different intensity of blue colour represents different ensemble member simulations.

Metadata: Marine heatwaves exacerbate climate change impacts for fisheries in the northeast Pacific

The dataset can be accessed at the online data repository *Dryad*: doi:10.5061/dryad.fj6q573qz . LME: EBS – Eastern Bering Sea, GoA – Gulf of Alaska, CC – California Current

| Dataset | File name | Variables |
| --- | --- | --- |
| Annual average sea surface temperature in each Large Marine Ecosystems | SST_Mean_[LME].csv | Year, Annual SST (^o^C), Smoothed annual SST (^o^C) |
| Projected Marine heatwave (MHWs) across the 10 ensemble member simulations | MHWs_SST.csv | LME, Ensemble member (1 to 10), Projected year of MHWs, SST anomalies relative to the decadal mean (^o^C) |
| Projected changes in mean annual biomass | MeanAbd_[LME]_[Species name].csv | Year, changes in biomass relative to the average of 1981-2000 (%), smoothed relative change in biomass relative to 1981-2000 (%) |
| Projected changes in mean latitudinal centroid | MeanLat_[LME]_[Species name].csv | Year, changes in latitudinal centroid relative to the average of 1981-2000 (km), smoothed changes in latitudinal centroid relative to the average of 1981-2000 (km) |
| Projected changes in mean bathymetry centroid | MeanBathy_[LME]_[Species name].csv | Year, changes in bathymetry centroid relative to the average of 1981-2000 (m), smoothed changes in bathymetry centroid relative to the average of 1981-2000 (m) |
| Projected changes in mean annual biomass during MHWs | MHWsAbd_[LME]_[Species name].csv | Year, changes in biomass relative to the decadal mean (%) |
| Projected changes in mean latitudinal centroid during MHWs | MHWsLat_[LME]_[Species name].csv | Year, changes in latitudinal centroid relative to the decadal mean (%) |
| Projected changes in mean bathymetry centroid during MHWs | MHWsBathy_[LME]_[Species name].csv | Year, changes in bathymetry centroid relative to the decadal mean (%) |
